# Sea Bass Fish Head Broth Treated by Thermo-Ultrasonication: Improving the Nutritional Properties and Emulsion Stability

**DOI:** 10.3390/foods13162498

**Published:** 2024-08-08

**Authors:** Huanqing Lei, Xinling Liu, Wei Zhao, Songyi Lin, Jiawei Lin, Jian Li, Xinan Zeng, Zhong Han

**Affiliations:** 1 School of Food Science and Engineering, South China University of Technology, Guangzhou 510641, China; leihuanqing@hotmail.com (H.L.); xinling_xenia@163.com (X.L.); jwlin.sci@hotmail.com (J.L.); 2 State Key Laboratory of Food Science and Technology, School of Food Science and Technology, Jiangnan University, Wuxi 214122, China; zhaow@jiangnan.edu.cn; 3 National Engineering Research Center of Seafood, School of Food Science and Technology, Dalian Polytechnic University, Dalian 116034, China; linsongyi730@163.com; 4 Guangdong Provincial Key Laboratory of Intelligent Food Manufacturing, Foshan University, Foshan 528225, China; li_jian@fosu.edu.cn (J.L.); xazeng@scut.edu.cn (X.Z.); 5 Preparatory Office, Yangjiang Applied Undergraduate College, Yangjiang 529500, China; 6 Overseas Expertise Introduction Center for Discipline Innovation of Food Nutrition and Human Health (111 Center), Guangzhou 510641, China

**Keywords:** sea bass fish head broth, thermo-ultrasonic treatment, nutritional properties, emulsion stability

## Abstract

This work investigated the underlying mechanism of thermo-ultrasonic treatment to improve the nutritional properties and emulsion stability of sea bass fish head broth. The effects of ultrasonication on the processing of fish broth were compared with boiling water treatment. The nutritional properties of fish broth mainly include protein, fat, total sugar, 5′-nucleotide and free amino acid content. To achieve a similar effect of nutrient extraction, the thermo-ultrasonic treatment required a shorter time (30 min) than boiling water (120 min). The water-soluble protein, fat and total sugar contents were at their maximum at 120 min of the thermo-ultrasonic treatment. In particular, the fat content increased with the time of thermo-ultrasonic treatment from 0.58% to 2.70%. The emulsion structure of the fish soup was characterized by measuring its color and particle size, using optical microscopy and confocal laser scanning microscopy, and determining its storage stability. Thermo-ultrasonic treatment reduced the particle size of the fish broth emulsion and the fat globules became smaller and more homogeneous. Ultrasonication not only accelerated the nutritional and flavor content of the fish head broth, but also reduced the particle size and enhanced the stability of the emulsified system of the fish broth. The fish head tissue was more severely disrupted by the cavitation effect of an ultrasound, and nutrients migrated more and faster. This was mainly due to the cavitation and mechanical breaking force of the ultrasound on the fish head tissue and the fat globules of the fish broth. Altogether, these findings suggest that the thermo-ultrasonic treatment technique is useful for processing nutrient-rich, storage-stable and ready-to-eat fish head broth.

## 1. Introduction

Broth is a very common food in the Chinese daily diet, which allows easy accessibility of nutrients. Broths made from different ingredients have different benefits and play an important role in human health due to their different nutrient composition [1]. Their characteristics of being nutritious and fresh-tasting, coupled with the ease in consumption of broths have led to their popularity since ancient times [2]. Broth from animals almost always contains proteins, lipids, sugars, amino acids, nucleotides and other minerals [3]. As a sea fish is rich in proteins, amino acids and unsaturated fatty acids [4], sea fish broth is highly nutritious and very important for maintaining a healthy diet. Fish protein is easily digested and rich in essential amino acids [5]. Studies have shown that fish protein hydrolysates, especially dipeptides and tripeptides, have antioxidant properties, antihypertensive, anticancer, anti-inflammatory and antibacterial effects [6,7]. Meanwhile, fish broth can be regarded as a complex colloidal system.

Sea bass is an economic fish from Southeast Asia that contains high levels of protein, essential amino acids and polyunsaturated fatty acids [8]. However, fish are easily spoiled and are difficult to preserve. For this reason, the development of product processing technology for sea bass broth has become vital [9]. Currently, sea bass broth is often used as a nutritional supplement for pregnant women, the elderly and frail post-operative patients to strengthen their wellbeing [10]. With the development of ready-to-eat foods and fast-paced lifestyles, ready-to-eat fish broths are chosen by many consumers. However, ready-to-eat fish head broth suffers from a low processing efficiency and poor product stability [11,12].

Ultrasonic technology is one of the most important methods for preparing emulsions [13], and in recent years, ultrasonic-assisted stewing has been developed in meat processing. Ultrasonic processing technology is a non-thermal treatment technology that plays a significant role in controlling, improving and accelerating processes [14]. Ultrasonic processing equipment generates ultrasonic waves, and the medium undergoes compression and expansion cycles at alternating high and low pressures [15]. The action of an ultrasound disrupts the tissue of food materials and accelerates the release, exchange and reaction of substances [16]. Traditional boiling is mainly a function of the transfer of thermal energy. There are reports on combining thermal and ultrasonic waves to improve food processing technology by utilizing the combined effects of cavitation and thermal energy [17]. In most of these studies, ultrasonic pre-treatment of food ingredients was mainly used before boiling. In the study by Jia et al., chicken broth was pre-treated under ultrasonic conditions at 600 W for 30 min, and they showed that the proportion of sweet and fresh amino acids in the broth increased, and the overall taste and flavor of the broth was enhanced [18]. The results by Wu et al. showed that chicken broth treated with 180 W of ultrasound combined with 0.1% (*w*/*w*) NaHCO_3_ had better flavor and sensory evaluation [19]. There have also been some studies conducted using thermo-ultrasonic food processing techniques. Han et al. showed that boiling bone broth for 1 h followed by thermo-ultrasonic for 10 min resulted in the highest protein and total sugar content, the smallest particle size and the richest content of the flavor substances [20]. Qi et al. showed that thermo-ultrasonic treatment could enhance the aromatic intensity of chicken broth by increasing its fat content and emulsion stability [21]. However, studies on the effects of the nutrients and stability of the emulsion in thermo-ultrasonic-treated fish broth and the correlation between the two properties are uncommon.

The experiments in this work were designed to study the effect of thermo-ultrasonic treatment on the nutritional and emulsion characteristics of sea bass fish head broth and to further elucidate the correlation between the two properties. Measurements were undertaken of the water-soluble protein, fat, total sugar, 5′-nucleotides, free amino acids (FAA), color, particle size, storage stability and changes in the emulsion structure in the fish head broth under optical microscopy and confocal laser scanning microscopy. We hope this study can provide some references for the processing method of ready-to-eat fish head broth dishes.

## 2. Materials and Methods

### 2.1. Materials and Chemicals

Fresh sea bass heads were purchased from the Guangzhou Wushan seafood market (Guangzhou, China) and stored at −20 °C. The oy bean oil used was the product of Jinlongyu Cereals and Oils Foodstuffs Co. (Shanghai, China). Adenosine 5′-monophosphate (5′-AMP), inosine 5′-monphosphate (5′-IMP) and guanosine 5′-monophosphate (5′-GMP) standards were obtained from Shanghai Macklin Biochemical Co., Ltd. (Shanghai, China). Folin–Ciocalteu’s phenol reagent, bovine serum albumin, NaOH, Na_2_CO_3_ and other unmentioned reagents were of analytical grade.

### 2.2. Preparation of Sea Bass Fish Head Broth

The preparation method of the sea bass fish head broth followed the method of Lin et al. with appropriate modifications [22]. Sea bass fish heads were cut into small pieces and ground using a grinder. The ground fish head was then fried with soy bean oil at a ratio of 20:1 and at 2100 W of frying for 40 s (the temperature of the oil was 120 °C). After frying, water was added to subsequently boil the fried ground fish with a fish to boiling water ratio of 1:4. The fish head broth was then packed separately in high-temperature cooking bags. All the samples were divided into two groups: one group was for boiling water (100 °C) treatment (Group A) and the other group was for thermo-ultrasonic treatment (Group B). Ultrasonic treatment was performed with the ultrasound-assisted boiling pot (THC-1000SF; Jining Tianhua Ultrasonic Electronic Instrument Co., Ltd., Jining, China). The ultrasound frequency of the equipment was 24.5 KHz, and the container size was 280 mm × 280 mm × 280 mm. The conditions of ultrasonic treatment were 500 W and 95 °C. The samples were boiled for 0 min (A1 and B1), 30 min (A2 and B2), 60 min (A3 and B3), 90 min (A4 and B4) and 120 min (A5 and B5). The samples were filtered through a double-layered medical sterile gauze and stored at 4 °C before analysis.

### 2.3. Determination of the Nutritional Composition in the Fish Broth

#### 2.3.1. Water-Soluble Proteins

The water-soluble protein content of the fish broth was determined by the Folin–Ciocalteu method, following the method of Qian et al., with appropriate modifications [23]. Specifically, the fish broth sample (1 mL) and Folin–Ciocalteu A solution (5 mL) were mixed well and reacted for 10 min at 25 °C. The Folin–Ciocalteu reagent (0.5 mL) was then added and mixed well immediately and then the mixture was water bathed at 30 °C for 30 min. Absorbance values were determined at 750 nm using an enzyme labeler (EPOCH12; BioTek Instruments. Inc., Winooski, VT, USA), with the bovine serum albumin solution (25, 50, 100, 150, 200, 250 μg/mL) as the standard samples. The water-soluble protein content was calculated from linear regression.

#### 2.3.2. Fat

The total fat of the fish broth samples was extracted by the chloroform/methanol method, referring to the method of Poon et al., with appropriate modifications [24]. Specifically, fish broth samples (20 g) were mixed with chloroform/methanol solution (100 mL, 2:1, *V*/*V*). After the sample was immersed for 24 h, 30 mL of 0.6% MgCl_2_ solution was added and left for a certain period of time to be stratified, and the upper layer of liquid was discarded to obtain the lower layer of fat extract. The extract was concentrated by rotary evaporation at 45 °C and dried to a constant weight to obtain the total fat of the fish broth.

#### 2.3.3. Total Sugar

The total sugar content was determined by the method of sulfuric acid-anthrone [25]. Fish broth samples (1 mL) were mixed with anthrone reagent (4 mL) and heated in a water bath at 95 °C for 15 min. After the samples were cooled in ice water, the absorbance values of the samples were analyzed at a wavelength of 620 nm using an enzyme labeler (EPOCH12; BioTek Instruments. Inc., Winooski, VT, USA).

#### 2.3.4. Nucleotides

The 5′-nucleotides were determined using the method of Kong et al., with some modifications [26]. Fish broth samples were mixed with 0.01 mol/L oxalic acid solution in a 1:1 ratio and centrifuged at 8000 rpm for 30 min. The supernatant was filtered using a 0.22 μm filter membrane and analyzed using high-performance liquid chromatography (HPLC, Hitachi L-8900; Hitachi Ltd., Tokyo, Japan). The model of the chromatography column was Agilent ZORBAX Eclipse XDB-C18 Agilent Ltd., Santa Clara; USA), 4.6 mm × 250 mm, 5 μm. The mobile phase was 0.2 mol/L KH_2_PO_4_ solution with a flow rate of 1.0 mL/min. The injection volume was 20 μL and the column temperature was 25 °C. The nucleotides were detected at 254 nm. 5′-GMP, 5′-IMP and 5′-AMP standard curves were used for the characterization and quantification of the nucleotides.

#### 2.3.5. Free Amino Acids

The free amino acids content was determined following the method of Kim et al., with some modifications [27]. Fish broth samples were mixed with 15% sulfosalicylic acid in a 4:1 ratio and the proteins were precipitated for 1 h at 4 °C. The samples were centrifuged twice at 10,000 rpm for 15 min and the sample supernatant was filtered using a 0.22 μm filter membrane. The samples were analyzed using an amino acid analyzer (Hitachi L-8900; Hitachi Ltd., Japan).

### 2.4. GC-MS Analysis

The flavor of sea bass fish broth was determined on a GC-MS system (GC-MS 8890-7000D; Agilent Ltd., Santa Clara, CA, USA) with an HP-WAX column (60 m × 20 mm × 20 μm). A head space solid-phase microextraction (HS-SPME) device with a 50/30 µm DVB/CAR/PDMS fiber was employed to equilibrate at 60 °C for 30 min and thermally desorb at 250 °C for 5 min. A total of 5 g of the fish soup samples was added into 20 mL headspace vials separately with 10 μL of 2-octanol solution (100 mg/L methanol solution). The temperature rise program was set to firstly increase the temperature at 45 °C and hold for 3 min, and to subsequently increase at 5 °C/min to 120 °C and hold for 3 min. The final sequence was to increase at 6 °C/min to 230 °C and hold for 5 min. The carrier of the system was helium, with a flow rate of 1 mL/min. The MS conditions were operated in the electron impact mode with 70 eV and a mass scan range with 35–350 amu. Comparative identification of volatile compounds was performed by comparing the mass spectral data with the NIST library and combining the results reported in the literature [28]. The concentration of volatile compounds was analyzed semi-quantitatively by comparing their peak areas with internal standards.

### 2.5. Emulsion Structure Characterization

#### 2.5.1. Color

A colorimeter (Ci7600; X-Rite, Grand Rapids, MI, USA) was used to determine the color values of the fish broth [20]. The instrument was calibrated using a white board and illuminant device. Lightness value (L*), redness value (a*) and yellowness value (b*) parameters were measured three times.

#### 2.5.2. Particle Size and Particle Size Distribution Range

The particle size and particle size distribution range of the emulsion within the fish broth samples were measured with a laser particle analyzer (Mastersizer 3000; Malvern Instruments Ltd., Malvern, UK), with water as the dispersed phase [29]. The shading ranges of the samples were 5–10%. The refractive indexes of the fish broth and dispersed phase were 1.46 and 1.33, respectively. The average particle size is expressed using d_3,2_ (surface area average particle size).

#### 2.5.3. Optical Microscopy

An amount of 20 μL of the fish broth samples was added onto microscopic slides and covered with coverslips. An optical microscope (DM750; Leica Instruments Co., Ltd., Wetzlar, Germany) with a 100× oil lens was used to observe and record the morphology of fish broth droplets.

#### 2.5.4. Confocal Laser Scanning Microscopy (CLSM)

The microstructure of the fish broth samples was observed using a confocal laser scanning microscope (Zeiss LSM800; Carl Zeiss, Jena, Germany) with a 63× oil lens. Nile blue and Nile red were used to stain the proteins (633 nm) and lipids (488 nm) of the fish broth samples, respectively. Approximately 20 μL of the sample was placed between the microscopic slide and the coverslip, making sure there were no air bubbles [30].

### 2.6. Storage Stability

A total amount of 10 mL of the fish broth samples was stored in clean serum bottles. Pictures of the different samples during the 7 d at 4 °C were obtained to observe the appearance and the status (stratification, flocculation, sedimentation and others) of the fish broth [31].

### 2.7. Statistical Analysis

The correlation between the fish broth components and the particle size was analyzed using Origin software (OriginPro Learning Edition; OriginLab Corp., Northampton, MA, USA). Experiments were performed in triplicate and statistical analysis was carried out using Origin software (OriginPro Learning Edition; OriginLab Corp., USA) and IBM SPSS Statistics 25 for variance analysis (ANOVA) and Duncan’s test (*p* < 0.05).

## 3. Results and Discussion

### 3.1. Changes in the Nutritional Composition of the Fish Broth

#### 3.1.1. Water-Soluble Proteins, Fats and Total Sugars

**Figure 1:**
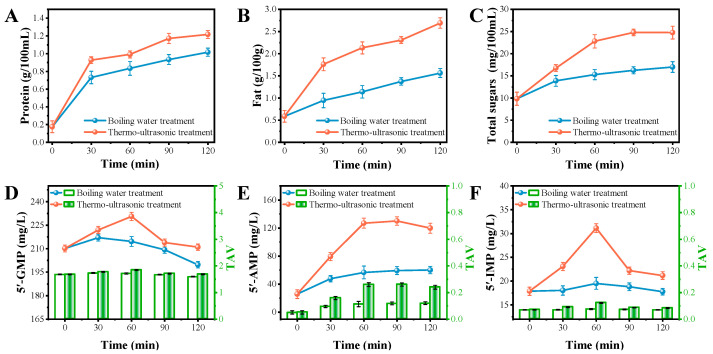
Changes in protein (**A**), fat content (**B**) and total sugar (**C**) by boiling water and thermo-ultrasonic treatment with different treatment times (0, 30, 60, 90, 120 min). The contents and TAV of 5′-GMP (**D**), 5′-AMP (**E**) and 5′-IMP (**F**) by boiling water and thermo-ultrasonic treatment with different treatment times (0, 30, 60, 90, 120 min).

Nutrients such as the proteins, fats and sugars in fish broth are important indicators of the flavor and nutrition of the broth. As shown in Figure 1A, the water-soluble protein contents increased over time in both the boiling water treatment and the thermo-ultrasonic treatment groups. The protein contents of the thermo-ultrasonic treatment group were significantly higher than in the boiling water group. The protein concentration for 30 min of thermo-ultrasonic treatment was 0.93 g/100 mL, which was similar to the result of 90 min of boiling water treatment. As shown in Figure 1B, the fat contents of the two groups increased as time went on, with the values for the thermo-ultrasonic treatment group (2.69 g/100 g) being larger than the boiling water group at 120 min (1.56 g/100 g). It is worth noting that the fat content of the thermo-ultrasonic treatment group reached 1.76 g/100 g at 30 min, which even exceeded the fat content of the boiling water group at 120 min. As shown in Figure 1C, the trend in the total sugar content was consistent with the protein. The total sugar content of the thermo-ultrasonic treatment group reached 24.74 mg/100 mL at 120 min. It is worth noting that the total sugar content of the thermo-ultrasonic treatment for 30 min was almost the same as that of the boiling water group for 120 min. Therefore, the fish broth processing method using thermo-ultrasonic treatment can reduce the extraction time of nutrients going into a broth. The results of nutrient extraction in the boiling water treatment group indicated that relatively high temperatures could promote the content and the nutrient extraction rate in fish broth processing, which is also known as the thermic effect [32]. By comparing the data from the two treatment groups, it was evident that thermo-ultrasonic treatment had a significant effect on the extraction of nutrients in fish broth processing. Han et al. [20] concluded that the shock waves and shear forces generated by an ultrasound can disrupt macromolecular nutrients in ground bone, allowing for an easier dissolution and delivery of nutrients.

#### 3.1.2. Nucleotides and Free Amino Acids

To analyze the effect of thermo-ultrasonic treatment on the taste of the fish broth, 5′-nucleotides (5′-GMP, 5′-IMP and 5′-AMP) were qualitatively and quantitatively quantified using HPLC. Nucleotides are important components in the formation of fish’s umami flavor, and there is a synergistic effect between nucleotides and flavor amino acids to produce a more intense umami flavor [33]. AMP and IMP are mainly produced by the degradation of adenosine triphosphate (ATP). While AMP promotes the perception of sweetness, IMP is an ideal flavor enhancer. GMP is a product of the conversion of IMP, which also possesses strong flavor-enhancing properties. As can be seen in Figure 1D–F, the thermo-ultrasonic treatment group had a higher content of all three 5′-nucleotides than the boiling water group at different times. The content of all three nucleotides from the fish broth that was thermo-ultrasonically treated for 60 min was the highest. Thermo-ultrasonic treatment effectively promotes the degradation of adenosine triphosphate (ATP) in fish heads, resulting in an elevated nucleotide content [34]. After thermo-ultrasonic treatment for more than 60 min, the content of 5′-GMP and 5′-IMP began to decrease, probably due to the occurrence of transformation or degradation [35]. The taste activity value (TAV) is often used to evaluate the taste contribution of compounds, and a TAV greater than 1 indicates that the TAV has a significant effect on food taste [17]. The highest content of 5′-GMP and all TAV values exceeded 1 in the samples, which indicated that 5′-GMP played an important role in the taste of the fish broth. However, the TAV values of 5′-AMP and 5′-IMP were less than 0.3, which had weak effects on the flavor of the fish broth.

**Figure 2:**
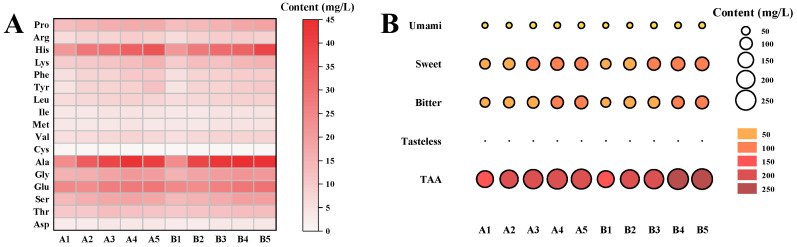
Heat map of the free amino acid content of fish broth (**A**) and the breakdown of the different types of free amino acids (**B**) by boiling water and thermo-ultrasonic treatment with different treatment times (0, 30, 60, 90, 120 min).

The results in Figure 2A show that free amino acids in the fish head tissue were gradually extracted into the broth as the processing time increased. Seventeen free amino acids were detected in both treatment groups, including seven essential and ten non-essential amino acids. The cavitation effect of the ultrasound and the denaturing effect of the thermal component of the treatment combined to accelerate the disruption of the fish head tissues. This accelerated the extraction of more free amino acids into the broth, resulting in an overall higher content of free amino acids in the thermo-ultrasonic treatment group. According to the study of Huang et al., free amino acids provide a variety of taste sensations including umami (Asp, Glu), sweetness (Ser, Pro, Gly, Thr, Ala), bitterness (Val, Met, Ile, Phe, Lys, Leu, Arg, His, Tyr) and tastelessness (Cys) [36]. From the results in Figure 2B, it can be seen that sweet amino acids were the main types of amino acids in the fish head broth. In particular, Ala, the most abundant amino acid in the broth, is a typically sweet amino acid. The total free amino acid and variously flavored amino acid contents of the thermo-ultrasonic treatment group were significantly higher than those of the boiling water group. The sweet amino acid content of B5 was 117.27 mg/L, which was much higher than the sweet amino acid content of A5 of 108.75 mg/L. However, the TAV values of all the types of flavored amino acids (Table S1) were much less than 1. In particular, the increase in the content of bitter amino acids did not have a significant effect on the flavor of the fish broth, which is the same as the findings of Zhu et al. [17].

### 3.2. Analysis of Volatile Compounds

Thirty-four different volatile compounds were identified in the boiling water treatment and the thermo-ultrasonic treatment groups of fish broths. Aldehydes and alcohols are the main volatile substances in sea bass fish head broth, while others are less abundant in the broth. Aldehydes are obtained by oxidative degradation of fatty acids, and Table 1 shows that a variety of aldehydes were detected in the fish head broth prepared by both processing methods. The main aldehydes are hexanal, heptanal, octanal and nonanal, and the related studies have shown that low-carbon aldehydes contribute more to the flavor of fish soup. Hexanal has a raw, greasy and grassy aroma, heptanal has a fruity aroma, octanal has a sharp and powerful tallowy-waxy aroma, and nonanal has a strong greasy odor and a sweet orange smell [37]. For example, the content of hexanal in the fish broth of the thermo-ultrasonic treatment group was almost twice as much as that of the boiling water group, which suggests that ultrasound contributes to the formation of flavor in the fish broth. The increase in the concentration of hexanal with time was due to the continuous oxidation of fat. However, it decreased at 120 min of heating, probably due to the formation of micro-nanostructures in the broth emulsion system. Alcohols are produced by redox reactions between polyunsaturated fatty acids and carbonyl compounds, and 12 types of alcohols were detected in the fish head broth. It has been reported in the literature that 1-octen-3-ol contributes to the overall flavor of fish head broth, which has a mushroom and fermented smell to enhance the fat flavor of fish soup. Other substances were also detected in the fish head broth, including 2-pentylfuran, which is an important flavoring substance produced during the processing of fish. Pathways for the production of 2-pentylfuran in broth include the Maillard reaction, carbohydrate degradation and lipid oxidation. The 2-pentylfuran content was higher in the thermo-ultrasonic treatment groups due to higher fat extraction. Therefore, the cavitation effect of ultrasound caused more fat from the fish head to migrate into the broth, thus increasing the concentration of the main characteristically flavored substances of the broth. Liu et al. [38] found that the intensity of freshness, richness and saltiness of fish bone broth can be enhanced by ultrasonic-assisted cooking technology, and the intensity of sourness reduced. The content of flavor nucleotides as well as fresh amino acids in fish bone broth increased, and the flavor quality of salmon bone broth improved. Thermo-ultrasonic technology may contribute to better flavor development in fish broth.

In summary, thermo-ultrasonic technology can promote faster and greater migration of substances, especially proteins and lipids, from sea bass fish heads into broth. The contents of total sugars, nucleotides and free amino acids were enhanced to different degrees. At the same time, it has been documented that an ultrasound can increase the soluble peptide content and minerals of broths [18]. However, proteins and lipids are the key components for the stability of the emulsification system of fish soup, and proteins such as emulsifiers can stabilize the oil–water interface to form a more stable emulsification system. Therefore, we conducted structural measurements of the emulsion system to investigate the effect of thermal sonication on the fish stock emulsion.

### 3.3. Emulsion Structure

#### 3.3.1. Color of the Fish Broth

**Figure 3:**
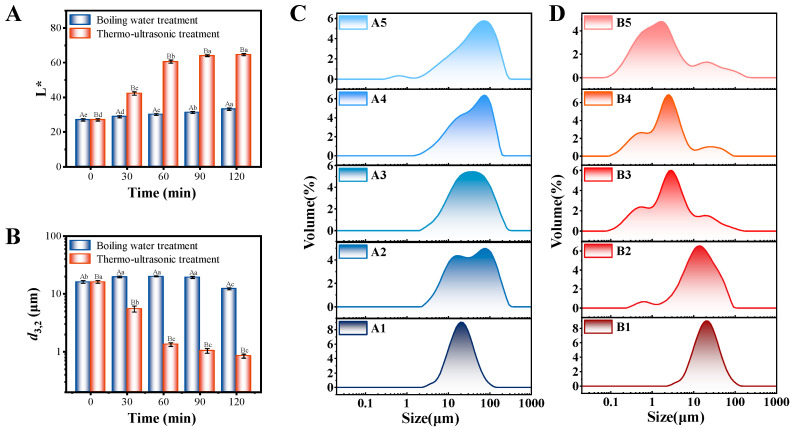
L* (**A**) and d_3,2_ (**B**) values of fish broth by different treatments and times. Droplet size distribution of fish broth by boiling water (**C**) and thermo-ultrasonic treatment (**D**) with different treatment times (1–5—0, 30, 60, 90, 120 min). The capital superscript letters (A and B) represent significant differences between treatment groups A and B, while small superscript letters (a, b, c, d, e) represent significant differences among treatment times (*p* < 0.05).

The effect of the thermo-ultrasonic treatment on the color of the broth is shown in Figure 3A. The L* value of the boiling water treatment group increased slowly with boiling time. The L* value of the thermo-ultrasonic treatment group gradually increased with boiling time and was significantly higher than that of the boiling water treatment group. Relatively, the a* and b* values (Figure S1) have a small range of variation and contributed less to the fish broth color. Therefore, the fish broth color depended mainly on the change in its brightness. The cavitation effect of thermo-ultrasonic treatment promoted the migration of nutrients into the broth at a faster time and in greater quantities. In particular, the dispersion of light was affected by the lipids, making the fish broth brighter and whiter [39].

#### 3.3.2. Particle Size Distribution Range

To evaluate the effect of thermo-ultrasonication on the stability of the fish broth, average particle size (d_3,2_) and particle size distribution were characterized. As shown in Figure 3B, under the thermo-ultrasonic treatment condition, d_3,2_ of the boiling water group showed a trend of increasing first and then decreasing. This phenomenon may be due to the fact that the nutrients were dissolved more and aggregated before 90 min, and the nutrient particles were dispersed after boiling for 120 min. The d_3,2_ of the thermo-ultrasonic treatment group decreased over time, with little change in values at 90 and 120 min. The results indicated that thermo-ultrasonic treatment had a positive effect on the particle size reduction in the fish broth. The particle size characteristic of the fish broth was visualized by the particle size distribution for different treatments and times. The results in Figure 3C show that the nanoscale particle size distribution appeared at 120 min in the boiling water group. The results in Figure 3D show that the distribution of nanoscale particles in the thermo-ultrasonic group started at 30 min and increased with longer treatment time. Yu et al. attributed the reduction in particle size to the ultrasonic effect induced by the collision of particles generating shock waves and to the collapse of cavitation bubbles in the solution [40]. Moreover, the thermal effect of boiling accelerates inter-particle collisions, achieving a synergistic effect with the ultrasound. The results indicated that the fish broth system was promoted to be more stable by the synergistic effects of the ultrasonic and thermal treatment.

#### 3.3.3. Optical Microstructure of the Fish Broth

**Figure 4:**
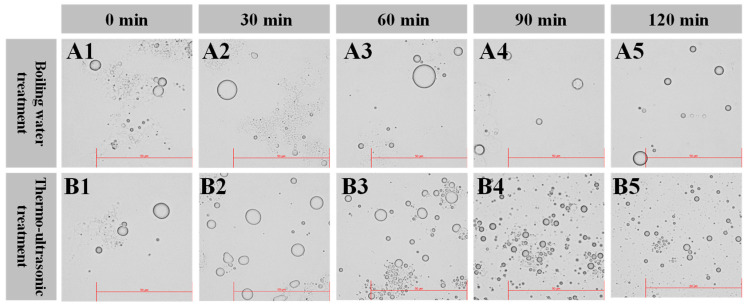
Optical micrographs of fish broth prepared by boiling water (**A**) and thermo-ultrasonic treatment (**B**) with different treatment times (**1**–**5**—0, 30, 60, 90, 120 min; Bar = 50 μm).

Figure 4 shows the micrographs of the different fish broth samples observed using an optical microscope. At 60 min, the fat droplets of the boiling water group became larger because of aggregation. At 120 min, the number of fat droplets became larger and the diameter eventually became smaller due to the stirring and thermal effects of boiling water. Fat droplets in the thermo-ultrasonic treatment group became greater in number and smaller in size as the heating time increased. At 120 min, the fat droplets in the thermo-ultrasonic group became more homogeneous under the combined effects of ultrasonic and thermal treatment. The cavitation effect of the thermal ultrasound broke up the emulsion in the fish broth into smaller balls which promoted emulsification, resulting in a more homogeneous fish broth throughout the system [41].

#### 3.3.4. Confocal Laser Scanning Microscopy

**Figure 5:**
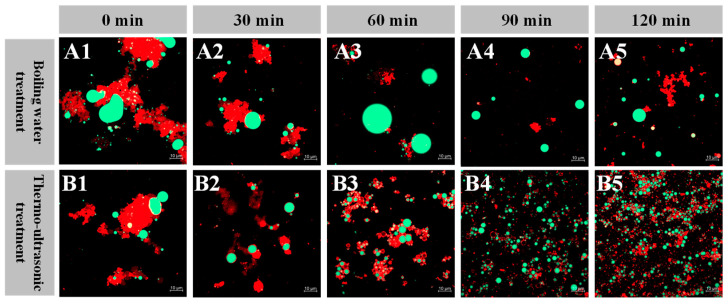
Confocal laser scanning micrographs of fish broth prepared by boiling water (**A**) and thermo-ultrasonic treatment (**B**) with different treatment times (**1**–**5**—0, 30, 60, 90, 120 min; Bar = 10 μm).

CLSM images of the fish broth are shown in Figure 5, with red and green fluorescence indicating the signals of protein and fat, respectively. The results of the CLSM showed a trend consistent with the optical micrographs. The fat droplets of the boiling water group first became bigger before 60 min, and then became progressively smaller. At subsequent times, the amount of fat and protein dissolved in the fish broth samples of the thermo-ultrasonic treatment group increased and dispersed more uniformly. The fat globules in the fish broth were surrounded by proteins. The longer the thermo-ultrasonic treatment was applied, the more nutrients were present in the emulsified system of the fish broth, which also became homogeneous and stable. Generally, proteins are amphiphilic emulsifiers with hydrophilic and hydrophobic regions. Proteins are adsorbed at the oil–water interface to form a stable interfacial film, which can reduce interfacial tension to stabilize a fish broth emulsification system [42]. The storage state of the fish broth samples with thermo-ultrasonic treatment for 120 min was the most stable.

#### 3.3.5. Storage Stability

**Figure 6:**
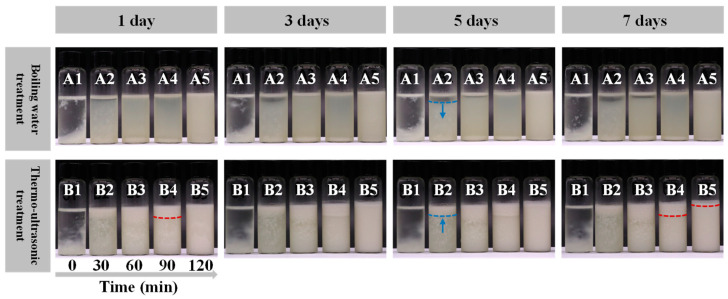
Appearance of the fish broth after storage at 1, 3, 5, 7 days by boiling water (A) and thermo-ultrasonic treatment (B) with different treatment times (1–5—0, 30, 60, 90, 120 min).

As shown in Figure 6, two treatment groups of the fish broth samples were stored for 1, 3, 5 and 7 days at 4 °C. The appearance and the status (stratification, flocculation, sedimentation, upward and other fluctuation) of the fish broths reflect the physical stability of the samples during storage. Flocculation and sedimentation were observed in groups A1 and B1 on Day 1. The reason for this phenomenon may be that less protein was dissolved in the fish broth at the beginning of the boiling process and the lack of tumbling action of the boiling water resulted in a weak emulsification of the fish broth. Samples from groups A2 and A3 showed significant stratification with extended storage time, while A4 and A5 showed a more stable emulsion system. This was probably due to the formation of an emulsified system. However, in order to develop nutritious and healthy fish broth products, the thermo-ultrasound group had significantly more nutrients in the fish broth than the boiling water group (Figure 1 and Figure 2). Samples B2, B3 and B4 of the thermo-ultrasonic treatment group showed varying degrees of fat uplifting during storage. Group B5 samples were stable on the first day with no fat uplifting, but slowly showed a small degree of fat uplifting in storage afterward. The storage state of the samples indicated that the emulsified system of the fish broth treated with thermo-ultrasonic treatment for 120 min was the most stable and its nutrient content was the highest. Increased total protein content promotes increased surface protein loading, which can affect emulsion stability. Broths treated with thermo-ultrasonic for 120 min had the highest protein content (Figure 1A), which enhanced the steric hindrance between oil droplets [17]. This may be one of the reasons for the higher stability of the broth samples that were thermo-ultrasonically treated for 120 min. In summary, the thermo-ultrasonic treatment method promoted better nutrients and stability in the fish broth, in support of the development of ready-to-eat fish broth products.

### 3.4. Correlation Analysis

**Figure 7:**
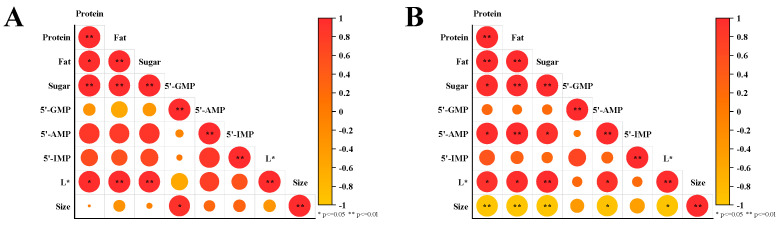
The correlation analysis of substances and droplet sizes of fish broth prepared by boiling water (**A**) and thermo−ultrasonic treatment (**B**) with different treatment times (0, 30, 60, 90, 120 min).

In order to analyze the correlation between the fish broth’s components and its particle size, the thermo-ultrasonic and boiling water treatment groups were analyzed (Figure 7). For both the A and B treatment groups, there was a positive correlation between protein, fat, total sugar and L* value, indicating that both thermal treatments alone and the synergistic effects of thermal and ultrasonic treatment can destroy fish head tissues. Various nutrients in the fish head tissues were gradually extracted into the broth with the same increasing trend. The dissolution of substances greatly changed the color characteristics of the fish head broth, especially the brightness. For the thermo-ultrasonic treatment group, the particle size was negatively correlated with protein, fat, total sugar, 5′-AMP and L* value, while there was no correlation between these indexes in the boiling water group. These results suggest that the effect of thermal and ultrasound treatment promotes nutrient dissolution, while the cavitation effect of the ultrasound makes the particle size of fish broth smaller, which ultimately leads to a more homogeneous and stable emulsification system of the fish broth. On the other hand, there was no correlation between these indexes in the boiling water heating group, which may be because only the effect of the thermal treatment was not enough to dissolve enough proteins and lipids to form small and stable oil droplets.

### 3.5. Impact of the Thermo-Ultrasonic Treatment on the Fish Broth Properties

To investigate the impact of thermo-ultrasonic treatment on the fish broth properties, we compared the nutrient and structural properties of the emulsion system of the fish broths by boiling water and thermo-ultrasonic treatment. Thermo-ultrasonic treatment accelerated the rate and the amount of nutrients (water-soluble proteins, fats and total sugars) extracted from the fish heads. When the thermo-ultrasonic treatment was undertaken for 30 min, the amount of nutrients extracted was close to or greater than the boiling water treatment at 120 min. The results from the structural characterization of the emulsified system of the fish broth showed that although thermo-ultrasonic treatment for 30 min led to the formation of an obvious emulsified system of the fish head broth, the fat uplifting phenomenon still existed, and the stability of the fish head broth product was poor. However, thermo-ultrasonic treatment for a longer period of time improved the stability of the fish broth.

**Figure 8:**
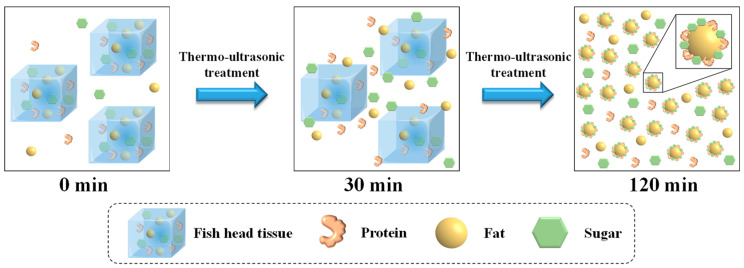
Schematic diagram of the thermo-ultrasonic treatment on fish broth.

Altogether, the impact mechanism of thermo-ultrasonic treatment on the fish broth properties is illustrated in Figure 8. Due to the destruction of the fish head tissue by thermal and ultrasonic effects for 30 min, greater quantities of nutrients such as proteins, fats and total sugars were extracted more rapidly into the broth to form an emulsified system (Figure 1A–C). The cavitation effect of sonication caused the fat globules in the fish broth to become smaller at 120 min, resulting in better stability of the fish broth emulsification system (Figure 3, Figure 4, Figure 5 and Figure 6). On the one hand, the cavitation and mechanical breakdown of an ultrasound can cause fish head tissue to be destroyed, accelerating the dissolution of nutrients. On the other hand, continuous ultrasonic treatment can break up a larger fat globule into smaller and more uniform droplets, which leads to an increase in the emulsification stability of a fish broth. Therefore, thermo-ultrasonic processing technology is an effective method that can enhance the rate and content of nutrient dissolution and also the stability of fish head broth.

## 4. Conclusions

The present work showed that thermo-ultrasonic treatment can improve the nutritional properties and emulsion stability of sea bass fish head broth. Short-term thermo-ultrasonic treatment can primarily increase the rate and amount of extraction of proteins, fats and total sugars. Nutrient concentration of a boiling water bath for 120 min can be achieved by thermal sonication for 30 min. The concentration of hexanal, nonanal, 1-octen-3-ol and 2-pentylfuran in the thermo-ultrasonic treatment groups was higher than the boiling water groups. Further, thermo-ultrasonic treatment can reduce the particle size and improve the storage stability of fish head broth. This is mainly due to the cavitation and mechanical breaking force of an ultrasound on fish head tissue and the fat globules of fish broth. Therefore, we can use thermo-ultrasonic technology to develop ready-to-eat fish broth products with higher nutrient content and superior storage stability. Ready-to-eat fish broth products can provide a richer diet and as a source of nutrition for special groups such as pregnant women and the elderly. As the effects of seasonings such as salt and ginger were not considered in this study, in the future, we will pay more attention to the flavor and taste of ready-to-eat fish broth.

## Figures and Tables

**Table 1:** Changes in flavor with different treatments and times.

			Relative Content to Internal Standard (ng/g)								
No.	Chemical Name	CAS	A1/B1	A2	A3	A4	A5	B2	B3	B4	B5
1	Hexanal	66-25-1	104.50 ± 5.17 ^d^	162.19 ± 4.32 ^c^	165.36 ± 2.65 ^c^	169.26 ± 4.79 ^c^	93.79 ± 5.21 ^e^	255.71 ± 6.17 ^a^	257.4 ± 3.41 ^a^	259.92 ± 2.45 ^a^	198.85 ± 1.79 ^b^
2	Octanal	124-13-0	7.67 ± 0.05 ^a^	ND	1.93 ± 0.23 ^b^	ND	ND	ND	ND	ND	ND
3	Undecanal	112-44-7	2.71 ± 0.03 ^b^	ND	ND	ND	ND	ND	2.99 ± 0.04 ^b^	ND	4.37 ± 0.52 ^a^
4	1-Nonanal	124-19-6	25.66 ± 1.25 ^de^	25.47 ± 0.85 ^de^	23.90 ± 0.75 ^e^	24.57 ± 1.03 ^e^	19.9 ± 1.31 ^f^	35.71 ± 2.06 ^b^	41.08 ± 1.36 ^a^	29.22 ± 1.02 ^c^	27.69 ± 1.17 ^cd^
5	(E)-2-Octenal	2548-87-0	2.46 ± 0.01 ^a^	ND	ND	ND	ND	ND	ND	ND	ND
6	Heptaldehyde	111-71-7	1.11 ± 0.04 ^b^	ND	ND	ND	ND	ND	ND	7.28 ± 0.85 ^a^	6.86 ± 0.84 ^a^
7	Decyl aldehyde	112-31-2	ND	2.91 ± 0.07 ^c^	3.41 ± 0.05 ^b^	ND	ND	ND	ND	3.26 ± 0.24 ^b^	8.22 ± 1.43 ^a^
8	(E)-2-Heptenal	18829-55-5	1.73 ± 0.21 ^b^	1.65 ± 0.65 ^b^	1.86 ± 0.73 ^b^	1.93 ± 0.54 ^b^	1.70 ± 0.49 ^b^	2.94 ± 0.48 ^a^	3.28 ± 0.61 ^a^	3.59 ± 0.86 ^a^	3.05 ± 0.39 ^a^
9	(Z)-4-Decenal	21662-09-9	ND	ND	ND	ND	ND	2.21 ± 0.05 ^a^	ND	2.09 ± 0.02 ^a^	ND
10	4-Ethylbenzaldehyde	4748-78-1	ND	1.09 ± 0.03 ^c^	ND	ND	ND	1.69 ± 0.91 ^b^	2.25 ± 0.06 ^a^	2.45 ± 0.06 ^a^	1.74 ± 0.51 ^b^
11	(E)-2-Nonenal	18829-56-6	1.08 ± 0.54 ^b^	1.12 ± 0.45 ^b^	1.28 ± 0.32 ^b^	1.21 ± 0.22 ^b^	1.37 ± 0.08 ^a^	1.41 ± 0.28 ^a^	1.58 ± 0.51 ^a^	1.50 ± 0.39 ^a^	1.60 ± 0.14 ^a^
12	(E)-2-Dodecenal	20407-84-5	1.23 ± 0.61 ^b^	1.28 ± 0.21 ^b^	1.35 ± 0.18 ^b^	1.38 ± 0.19 ^b^	1.50 ± 0.14 ^a^	1.58 ± 0.35 ^a^	1.68 ± 0.24 ^a^	1.75 ± 0.18 ^a^	1.97 ± 0.38 ^a^
13	3-Heptylacrolein	3913-81-3	ND	ND	0.62 ± 0.01 ^b^	ND	ND	ND	ND	1.36 ± 0.04 ^a^	ND
14	Dodecyl aldehyde	112-54-9	ND	ND	0.72 ± 0.03 ^b^	0.82 ± 0.02 ^b^	ND	ND	ND	1.22 ± 0.02 ^a^	1.64 ± 0.33 ^a^
15	Benzaldehyde	100-52-7	7.08 ± 0.39 ^c^	7.75 ± 0.65 ^c^	9.55 ± 0.12 ^b^	10.93 ± 0.85 ^a^	ND	9.99 ± 0.64 ^ab^	9.49 ± 0.93 ^b^	9.16 ± 0.54 ^b^	7.94 ± 0.63 ^c^
16	(E,E)-2,4-Nonadienal	5910-87-2	0.79 ± 0.05 ^bc^	0.83 ± 0.15 ^b^	0.78 ± 0.16 ^c^	0.85 ± 0.01 ^b^	0.90 ± 0.10 ^b^	0.92 ± 0.08 ^ab^	0.98 ± 0.05 ^a^	1.15 ± 0.11 ^a^	1.28 ± 0.13 ^a^
17	(E,E)-2,4-Decadienal	25152-84-5	4.45 ± 0.09 ^c^	2.40 ± 0.08 ^e^	3.28 ± 0.09 ^d^	2.78 ± 0.06 ^d^	ND	8.77 ± 0.35 ^b^	13.25 ± 1.01 ^a^	7.6 ± 0.65 ^b^	5.51 ± 0.25 ^c^
18	Isododecyl alcohol	3913-02-8	ND	3.58 ± 0.04 ^d^	ND	ND	27.06 ± 2.13 ^b^	5.46 ± 0.25 ^d^	67.43 ± 2.37 ^a^	11.78 ± 0.21 ^c^	ND
19	DL-Menthol	89-78-1	ND	42.09 ± 1.19 ^c^	53.21 ± 1.02 ^b^	49.32 ± 0.69 ^b^	43.93 ± 1.95 ^c^	38.24 ± 3.10 ^c^	ND	60.79 ± 3.71 ^a^	ND
20	Phytol	150-86-7	ND	ND	7.60 ± 0.65 ^b^	7.46 ± 0.15 ^b^	10.64 ± 0.37 ^a^	ND	ND	ND	ND
21	1-Octen-3-ol	3391-86-4	12.48 ± 0.73 ^c^	20.64 ± 1.62 ^b^	22.95 ± 1.35 ^b^	21.27 ± 0.98 ^b^	ND	32.86 ± 1.26 ^a^	36.90 ± 2.54 ^a^	ND	29.52 ± 1.49 ^a^
22	3,4-Dimethylcyclohexanol	5715-23-1	ND	4.30 ± 0.01 ^a^	3.52 ± 0.32 ^b^	ND	ND	ND	2.30 ± 0.01 ^c^	ND	ND
23	2-Octyn-1-ol	20739-58-6	4.83 ± 0.24 ^a^	ND	ND	ND	ND	ND	ND	ND	ND
24	2-Decyn-1-ol	4117-14-0	ND	4.87 ± 0.02 ^a^	ND	ND	ND	ND	ND	ND	ND
25	1,7-octandiene-3-ol	30385-19-4	ND	ND	4.16 ± 0.72 ^c^	ND	ND	5.56 ± 0.18 ^a^	4.98 ± 0.07 ^b^	ND	ND
26	4-Ethylcyclohexanol	4534-74-1	ND	2.98 ± 0.01 ^c^	4.00 ± 0.24 ^b^	4.48 ± 0.23 ^b^	ND	ND	5.02 ± 0.16 ^a^	3.07 ± 0.08 ^c^	5.73 ± 0.49 ^a^
27	2,3-Dimethylcyclohexanol	1502-24-5	ND	1.31 ± 0.05 ^b^	ND	ND	1.79 ± 0.12 ^a^	ND	ND	ND	ND
28	(E)-2-Octen-1-ol	18409-17-1	3.16 ± 0.14 ^c^	3.28 ± 0.18 ^c^	3.31 ± 0.21 ^c^	3.42 ± 0.39 ^bc^	3.39 ± 0.52 ^bc^	3.89 ± 0.24 ^ab^	3.95 ± 0.16 ^a^	4.03 ± 0.28 ^a^	4.10 ± 0.21 ^a^
29	L-Menthol	2216-51-5	ND	ND	1.50 ± 0.05	ND	ND	1.20 ± 0.04	ND	1.10 ± 0.01	1.30 ± 0.62
30	P-Xylene	106-42-3	ND	8.67 ± 0.56 ^a^	ND	ND	ND	8.32 ± 0.36 ^a^	ND	ND	ND
31	2-pentylfuran	3777-69-3	6.61 ± 0.18 ^d^	7.85 ± 0.28 ^d^	18.08 ± 0.42 ^c^	48.36 ± 1.85 ^a^	40.93 ± 2.35 ^b^	24.68 ± 0.27 ^c^	36.94 ± 0.19 ^b^	45.69 ± 0.38 ^a^	52.89 ± 1.65 ^a^
32	Mesitylene	108-67-8	14.98 ± 0.95 ^a^	17.24 ± 0.72 ^a^	11.97 ± 2.30 ^b^	5.27 ± 0.35 ^c^	ND	ND	ND	ND	ND
33	2,6-Di-tert-butyl-4- methylphenol	128-37-0	46.95 ± 1.03 ^a^	45.39 ± 1.22 ^a^	46.36 ± 3.12 ^a^	43.18 ± 1.20 ^a^	27.55 ± 2.15 ^b^	21.26 ± 1.36 ^c^	30.01 ± 1.07 ^b^	19.09 ± 1.28 ^c^	24.97 ± 0.94 ^b^
34	2,4-Di-tert-butylphenol	96-76-4	ND	1.94 ± 0.01 ^b^	3.07 ± 0.52 ^a^	1.97 ± 0.03 ^b^	2.26 ± 0.05 ^b^	2.71 ± 0.54 ^a^	3.15 ± 0.15 ^a^	2.81 ± 0.42 ^a^	3.17 ± 0.05 ^a^

Small superscript letters (a, b, c, d, e, f) represent significant differences among treatment times (*p* < 0.05). “ND” represent not detected.

## Data Availability

The original contributions presented in the study are included in the article/Supplementary Materials, further inquiries can be directed to the corresponding author.

## Supplementary Materials

The following supporting information can be downloaded at: https://www.mdpi.com/article/10.3390/foods13162498/s1, Figure S1: The *a** and *b** values of fish broth by different treatment; Table S1: Free amino acids (FAAs) of the sea bass fish head broth.
