# Supplementary material for: Sea Bass Fish Head Broth Treated by Thermo-Ultrasonication: Improving the Nutritional Properties and Emulsion Stability
Source: Foods. 2024 Aug 8;13(16):2498. doi: 10.3390/foods13162498 (PMC11354003; doi:10.3390/foods13162498)
Supplement: Supplementary file 1 [file foods-13-02498-s001.zip › foods-3098263-supplementary.pdf]

Supplementary Materials

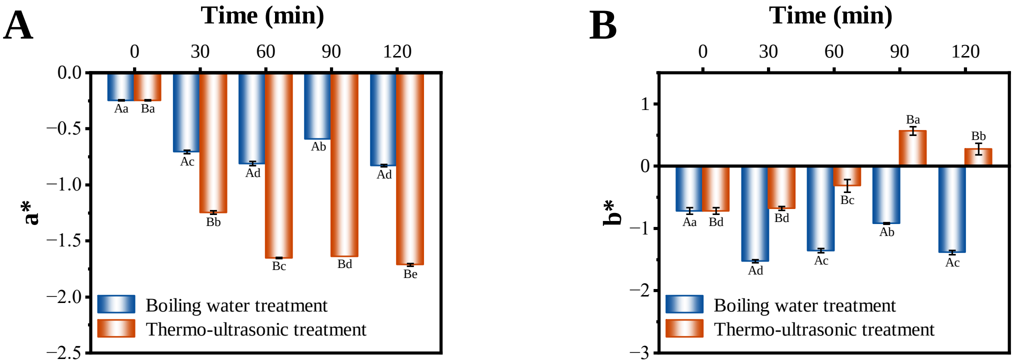

Figure S1. The a* and b* values of fish broth by different treatment.

Table S1. Free amino acids (FAAs) of the sea bass fish head broth.

FAAs	Threshold value (mg/L)	Time (min)	Boiling water treatment		Thermo-ultrasonic treatment	
			Content (mg/L)	TAV	Content (mg/L)	TAV
Asp	1000	0	2.768±0.029	0.003	2.768±0.029	0.003
		30	3.128±0.017	0.003	3.141±0.062	0.003
		60	3.527±0.051	0.004	3.554±0.055	0.004
		90	3.807±0.072	0.004	3.807±0.057	0.004
		120	3.501±0.043	0.004	3.487±0.021	0.003
Thr	2600	0	10.284±0.058	0.004	10.284±0.058	0.004
		30	10.200±0.050	0.004	11.535±0.072	0.004
		60	12.548±0.011	0.005	12.178±0.042	0.005
		90	12.309±0.047	0.005	13.227±0.060	0.005
		120	11.642±0.011	0.004	12.905±0.034	0.005
Ser	1500	0	13.777±0.170	0.009	13.777±0.170	0.009
		30	15.932±0.101	0.011	15.753±0.071	0.011
		60	17.760±0.047	0.012	17.298±0.183	0.012
		90	18.380±0.081	0.012	19.200±0.100	0.013
		120	18.286±0.153	0.012	19.830±0.054	0.013
Glu	300	0	24.482±0.213	0.082	24.482±0.213	0.082
		30	23.453±0.277	0.078	23.923±0.048	0.080
		60	27.351±0.155	0.091	26.336±0.283	0.088
		90	28.234±0.272	0.094	28.779±0.176	0.096
		120	28.749±0.037	0.096	29.853±0.238	0.100
Gly	1300	0	15.744±0.085	0.012	15.744±0.085	0.012
		30	16.560±0.023	0.013	19.144±0.057	0.015
		60	18.867±0.015	0.015	20.682±0.086	0.016
		90	20.933±0.043	0.016	21.716±0.062	0.017
		120	20.743±0.031	0.016	21.505±0.030	0.017
Ala	600	0	23.404±0.282	0.039	23.404±0.282	0.039
		30	34.415±0.182	0.057	39.262±0.284	0.065
		60	39.583±0.248	0.066	42.932±0.239	0.072
		90	44.091±0.167	0.073	44.830±0.113	0.075
		120	41.142±0.255	0.069	44.010±0.169	0.073
Cys	-	0	0.351±0.034	-	0.351±0.034	-
		30	0.315±0.018	-	0.315±0.023	-
		60	0.315±0.043	-	0.363±0.032	-
		90	0.376±0.020	-	0.376±0.031	-
		120	0.388±0.025	-	0.351±0.017	-
Val	400	0	5.811±0.070	0.015	5.811±0.070	0.015
		30	6.385±0.067	0.016	6.935±0.047	0.017

		60	7.287±0.080	0.018	7.509±0.040	0.019
		90	7.708±0.044	0.019	8.060±0.038	0.020
		120	7.228±0.052	0.018	7.837±0.019	0.020
		0	3.626±0.083	0.012	3.626±0.083	0.012
		30	3.581±0.039	0.012	3.715±0.037	0.012
Met	300	60	3.939±0.093	0.013	4.223±0.045	0.014
		90	4.252±0.089	0.014	4.536±0.067	0.015
		120	3.924±0.030	0.013	4.327±0.037	0.014
		0	3.738±0.036	0.004	3.738±0.036	0.004
		30	3.765±0.078	0.004	4.106±0.094	0.005
Ile	900	60	4.525±0.077	0.005	4.512±0.023	0.005
		90	4.617±0.064	0.005	4.827±0.034	0.005
		120	4.276±0.024	0.005	4.880±0.029	0.005
		0	5.942±0.182	0.003	5.942±0.182	0.003
		30	7.096±0.098	0.004	7.346±0.177	0.004
Leu	1900	60	7.975±0.031	0.004	8.237±0.024	0.004
		90	8.565±0.180	0.005	8.906±0.055	0.005
		120	7.988±0.083	0.004	8.893±0.096	0.005
		0	4.457±0.140	-	4.457±0.140	-
		30	7.574±0.104	-	8.009±0.093	-
Tyr	-	60	7.954±0.074	-	7.846±0.076	-
		90	8.951±0.028	-	9.821±0.078	-
		120	11.995±0.109	-	10.292±0.155	-
		0	5.550±0.127	0.006	5.550±0.127	0.006
		30	7.731±0.134	0.009	7.549±0.098	0.008
Phe	900	60	7.549±0.140	0.008	9.069±0.103	0.010
		90	10.027±0.053	0.011	9.366±0.077	0.010
		120	10.655±0.104	0.012	9.598±0.033	0.011
		0	9.385±0.215	0.019	9.385±0.215	0.019
		30	10.453±0.274	0.021	12.543±0.175	0.025
Lys	500	60	11.052±0.097	0.022	11.856±0.168	0.024
		90	13.303±0.078	0.027	14.444±0.277	0.029
		120	15.335±0.099	0.031	15.642±0.102	0.031
		0	21.443±0.147	0.107	21.443±0.147	0.107
		30	28.844±0.285	0.144	28.006±0.153	0.140
His	200	60	29.775±0.102	0.149	31.125±0.202	0.156
		90	33.965±0.167	0.170	33.127±0.099	0.166
		120	36.385±0.080	0.182	39.954±0.156	0.200
		0	6.027±0.177	0.012	6.027±0.177	0.012
Arg	500	30	8.048±0.135	0.016	8.501±0.163	0.017
		60	8.135±0.087	0.016	9.302±0.175	0.019
		90	9.355±0.074	0.019	9.250±0.181	0.019

		120	8.884±0.074	0.018	8.849±0.096	0.018
Pro	3000	0	13.332±0.024	0.004	13.332±0.024	0.004
		30	14.380±0.074	0.005	14.149±0.064	0.005
		60	16.452±0.058	0.005	15.623±0.028	0.005
		90	16.728±0.027	0.006	17.695±0.091	0.006
		120	16.936±0.066	0.006	19.019±0.026	0.006
Umami	-	0	27.251	-	27.251	-
		30	26.580	-	27.065	-
		60	30.879	-	29.890	-
		90	32.041	-	32.585	-
		120	32.250	-	33.340	-
Sweet	-	0	76.540	-	76.540	-
		30	91.487	-	99.843	-
		60	105.209	-	108.713	-
		90	112.441	-	116.669	-
		120	108.748	-	117.270	-
Bitter	-	0	65.980	-	65.980	-
		30	83.476	-	86.710	-
		60	88.192	-	93.680	-
		90	100.744	-	102.336	-
		120	106.671	-	110.272	-
Tasteless	-	0	0.351	-	0.351	-
		30	0.315	-	0.315	-
		60	0.315	-	0.363	-
		90	0.376	-	0.376	-
		120	0.388	-	0.351	-
TAA	-	0	170.123	-	170.123	-
		30	201.859	-	213.933	-
		60	224.595	-	232.646	-
		90	245.601	-	251.966	-
		120	248.057	-	261.233	-
